# Precision Nutraceuticals and Biomarkers of Healthy Aging: A Scoping Review of Human Studies on Nutrigenomic-Based Interventions

**DOI:** 10.3390/medsci14050508

**Published:** 2026-08-23

**Authors:** Andrea Vanessa Llanos-Díaz, Moisés Apolaya-Segura, Orlando R. Sevillano, Obert Marín-Sanchez, Jacinto Joaquín Vértiz Osores, Alexis Germán Murillo Carrasco, Sophie Nicole Llanos-Diaz, Daysi Zulema Diaz-Obregón

**Affiliations:** 1 Biology, Southern Utah University, Cedar City, UT 84720, USA; 2 ONG Innovation and Science for the Care and Support of Society–INNOVACARE, Lima 15023, Perudaysi.diaz@essalud.gob.pe (D.Z.D.-O.); 3 Collaborative Initiative for Public Health and Epidemiological Research in Latin America and the Caribbean (CIPHER-LAC Research Group), Universidad Científica del Sur, Lima 15067, Peru; 4 Instituto de Evaluación de Tecnologías en Salud e Investigación—IETSI—de EsSalud, Lima 15072, Peru; 5 Universidad Autónoma de Ica, Chincha Alta 11701, Peru; 6 Organization for Medical Innovation and Collaboration for Sciences—OMICS, Lima 15072, Peru; omarins@untels.edu.pe; 7 Grupo de Investigación en Bioprospección en Salud y Metagenómica Ambiental, Universidad Nacional Tecnológica de Lima Sur, Lima 15834, Peru

**Keywords:** nutrigenomics, precision medicine, dietary supplements, healthy aging, biomarkers

## Abstract

Background: Precision nutrition and nutrigenomics have emerged as promising strategies to personalize dietary interventions according to individual genetic, metabolic, and molecular characteristics. However, the potential of nutrigenomic-guided nutraceutical interventions to modulate biological aging pathways remains poorly characterized. Objective: This study aimed to map and synthesize the available evidence from human studies evaluating personalized nutraceutical interventions based on nutrigenomic approaches and their effects on biomarkers associated with biological aging. Methods: A scoping review was conducted following the Joanna Briggs Institute methodology and PRISMA-ScR guidelines. PubMed/MEDLINE, Embase, Scopus, and Web of Science were searched for studies published between 2021 and 2026. Eligible studies included adults aged ≥18 years receiving personalized nutraceutical interventions informed by genetic, genomic, metabolomic, or other molecular data. Outcomes included biomarkers of cellular aging, inflammation, oxidative stress, immunometabolism, and related pathways. Results: Twenty-one studies were included. Interventions involved omega-3 fatty acids, polyphenols, Nigella sativa, vitamin D, methyl-donor micronutrients, fermented papaya preparation, and other nutraceutical preparations and functional food-based interventions. Four major themes emerged: multi-omic stratification approaches, biological aging biomarkers, redox-inflammatory modulation, and applications in metabolic disorders. Most studies demonstrated favorable effects on intermediate molecular biomarkers, including inflammatory cytokines, oxidative stress markers, lipid metabolism, endothelial function, and DNA methylation signatures. However, evidence directly demonstrating slowing of biological aging through validated biomarkers such as epigenetic clocks or telomere dynamics remains limited. Conclusions: Personalized nutraceutical interventions exhibit biological plausibility for modulating pathways associated with healthy aging. Nevertheless, robust longitudinal studies incorporating validated biomarkers of biological aging and clinically meaningful outcomes are needed before their widespread implementation in precision medicine.

## 1. Introduction

Population aging has emerged as one of the most significant public health challenges of the twenty-first century, driven by substantial increases in life expectancy and demographic transitions occurring worldwide [1,2]. The extension of lifespan has been accompanied by a growing prevalence of age-related chronic conditions, including obesity, type 2 diabetes mellitus, cardiovascular diseases, frailty, neurodegenerative disorders, and metabolic syndrome, all of which contribute substantially to morbidity, disability, and healthcare expenditures among older adults [3,4]. These disorders share several biological mechanisms associated with aging, including chronic low-grade inflammation (inflammaging), oxidative stress, mitochondrial dysfunction, genomic instability, and cellular senescence, which collectively promote the progressive decline in physiological resilience and functional capacity observed with advancing age [5,6].

These mechanisms are encompassed within the hallmarks of aging framework, which includes genomic instability, epigenetic alterations, loss of proteostasis, mitochondrial dysfunction, stem cell exhaustion, and cellular senescence [5,6]. Senescent cells progressively accumulate in multiple tissues during aging and secrete a complex mixture of pro-inflammatory cytokines, chemokines, growth factors, and proteases collectively known as the senescence-associated secretory phenotype (SASP), which contributes to chronic inflammation, tissue dysfunction, and the pathogenesis of age-related diseases [7,8]. Consequently, biomarkers such as telomere length, telomerase activity, DNA methylation-based epigenetic clocks, expression of cell-cycle inhibitors including p16INK4A and p21, circulating inflammatory cytokines, and oxidative stress markers have emerged as valuable indicators of biological aging and age-related health status [6,9].

Nutritional interventions have been extensively investigated as potential strategies to modulate the biological processes underlying aging and age-related diseases [4,10]. In particular, bioactive compounds and food-derived constituents, including omega-3 fatty acids, polyphenols, resveratrol, curcumin, quercetin, antioxidant vitamins, and nicotinamide adenine dinucleotide (NAD^+^) precursors, have demonstrated anti-inflammatory, antioxidant, senomorphic, and mitochondrial-related effects in experimental and clinical studies [3,11]. Nevertheless, considerable interindividual variability has been observed in clinical responses to these interventions, suggesting that genetic background and molecular heterogeneity may substantially influence treatment efficacy and biological outcomes [12,13].

Recent advances in nutrigenomics and precision nutrition have enabled the development of personalized dietary and nutraceutical interventions based on individual genetic, metabolic, and molecular characteristics [12,13]. Nutrigenomics examines the effects of nutrients and bioactive compounds on gene expression and cellular function, whereas nutrigenetics investigates how genetic variants influence individual responses to dietary exposures [12,14]. The incorporation of genomic information, including single-nucleotide polymorphisms (SNPs) and other genetic markers, has facilitated the design of tailored nutritional strategies intended to optimize metabolic health, reduce chronic disease risk, and potentially influence biological aging trajectories [12,15].

Nutraceuticals and functional foods were considered related but distinct intervention categories. Nutraceuticals refer to concentrated food-derived preparations, supplements, or formulations intended to provide a health benefit, whereas functional foods are foods or food-based preparations that provide bioactive constituents in addition to their conventional nutritional value. Individual bioactive molecules or active constituents contained within these products were described as bioactive compounds rather than as nutraceuticals themselves.

Within this framework, precision nutraceuticals represent the intersection between nutraceutical interventions and precision nutrition, in which biological information is used to identify appropriate interventions, stratify individuals according to expected response, or characterize molecular response patterns. Importantly, not all nutraceutical interventions are precision interventions; the term “precision nutraceutical” is therefore reserved for interventions or evidence in which individual biological characteristics are explicitly connected to intervention selection, stratification, or response assessment.

Despite increasing scientific and commercial interest in precision nutrition, evidence regarding the effectiveness of nutrigenomic-guided nutraceutical interventions on biomarkers of cellular aging remains limited and fragmented [15,16]. Existing reviews have largely focused on metabolic outcomes, body weight regulation, cardiometabolic risk factors, and dietary personalization. In contrast, the specific effects of nutrigenomic-based interventions on biological aging markers, systemic inflammation, oxidative stress, mitochondrial function, and senescence-related pathways have not been comprehensively synthesized [12,15].

Therefore, this scoping review aims to map and summarize the available evidence from human studies evaluating nutrigenomic-based personalized nutraceutical interventions in adults aged 18 years and older. Specifically, this review aims to (1) map nutrigenomic and precision nutrition strategies used in personalized nutraceutical interventions; (2) summarize the aging-related biomarkers assessed; and (3) identify evidence gaps and future research priorities. By providing a comprehensive overview of the current evidence landscape, this review may help identify critical knowledge gaps and inform future clinical research on precision nutrition strategies for healthy aging and longevity.

## 2. Materials and Methods

### 2.1. Study Design

This scoping review was conducted to map and synthesize the available evidence regarding the effects of nutrigenomic-guided nutraceutical interventions on biomarkers of cellular aging in adults aged ≥18 years. The review followed the methodological framework proposed by Arksey and O’Malley and subsequent recommendations from the Joanna Briggs Institute (JBI) for scoping reviews [17]. Reporting was guided by the Preferred Reporting Items for Systematic Reviews and Meta-Analyses extension for Scoping Reviews (PRISMA-ScR) [18] and formally deposited to Open Science Framework (https://osf.io/gfsbd/overview?view_only=add911208eca439fa7d350de50a6cc8a, accessed on 19 August 2026). The OSF number is 10.17605/OSF.IO/QHRFW.

### 2.2. Research Question and Eligibility Criteria

The review question was developed according to the Population, Concept, and Context (PCC) framework recommended by the Joanna Briggs Institute for scoping reviews. For this study, we included the following components: Population (adults aged ≥18 years); Concept (nutrigenomic-based nutraceutical interventions, spanning genotype-guided, biomarker/metabotype-guided, and molecular-response approaches); Context (biological aging and age-related conditions), to respond to the following question: In adults aged 18 years and older, do personalized nutraceutical interventions based on nutrigenomic information, compared with standard or non-personalized nutritional interventions, improve biomarkers of cellular aging, systemic inflammation, and oxidative stress?

The review was initially designed to include adults aged ≥40 years. However, during full-text assessment, it became evident that the limited available evidence frequently involved broader adult populations, including participants younger than 40 years, who otherwise met the predefined eligibility criteria regarding study design, nutrigenomic intervention, and relevant outcomes. Given the exploratory and evidence-mapping purpose of this scoping review, the population criterion was therefore broadened to adults aged ≥18 years to avoid excluding otherwise eligible human studies solely on the basis of age range. This amendment was applied to the final eligibility assessment and is reported here to ensure methodological transparency.

For the final eligibility assessment, the population of interest included human adults aged 18 years or older, either healthy or presenting age-related chronic conditions associated with metabolic aging, including obesity, metabolic syndrome, type 2 diabetes mellitus, frailty, chronic low-grade inflammation, and healthy aging populations. We excluded animal and in vitro studies, studies involving participants younger than 18 years, non-original research articles, studies without a nutrigenomic-based nutritional or nutraceutical component, and studies that did not report outcomes relevant to the review objectives.

Eligible interventions consisted of personalized nutritional or nutraceutical strategies informed by genetic, genomic, or nutrigenomic information. Consistent with the mapping purpose of a scoping review, we operationalized the nutrigenomic concept at three levels: (i) genotype-guided interventions, in which supplementation or dietary advice was selected or stratified by specific genetic variants or single-nucleotide polymorphisms (SNPs); (ii) biomarker- or metabotype-guided interventions, tailored according to baseline metabolic, inflammatory, or multi-omic profiles; and (iii) studies characterizing the molecular or nutrigenomic response to a bioactive compound (e.g., gene-expression, epigenetic, or metabolomic readouts), even when the intervention itself was not individually tailored. This inclusive operationalization was adopted to capture the full landscape of nutrigenomic-based evidence relevant to biological aging.

Comparator groups included standard dietary recommendations, conventional nutritional interventions, non-personalized supplementation protocols, placebo, or usual care. In accordance with the exploratory nature of scoping reviews, studies lacking a formal comparator group were also considered when they provided relevant information regarding nutrigenomic-guided interventions and aging-related outcomes.

### 2.3. Outcomes of Interest

The primary outcomes of interest were biomarkers related to cellular and biological aging, including validated measures of biological age when available (e.g., epigenetic clocks or composite biological-age measures), as well as aging-related surrogate biomarkers such as telomere length, telomerase activity, senescence-related markers including p16INK4A and p21, DNA methylation measures, and senescence-associated secretory phenotype (SASP) biomarkers.

Secondary outcomes included markers of systemic inflammation, such as C-reactive protein (CRP), interleukin-6 (IL-6), tumor necrosis factor alpha (TNF-α), and interleukin-1β (IL-1β), as well as oxidative stress biomarkers including malondialdehyde (MDA), reactive oxygen species (ROS), superoxide dismutase (SOD), glutathione peroxidase (GPx), catalase activity, and total antioxidant capacity (TAC). Additional outcomes related to mitochondrial function, DNA damage, and immunosenescence were also considered when available.

### 2.4. Information Sources and Search Strategy

A comprehensive search strategy was developed to identify relevant studies published over the last six years (2021–2026) in PubMed/MEDLINE, Embase, Scopus, and Web of Science. The search combined controlled vocabulary terms and free-text keywords related to nutrigenomics, nutrigenetics, precision nutrition, personalized nutrition, nutraceuticals, aging, cellular senescence, inflammation, and oxidative stress. Representative search terms included combinations of “nutrigenomics”, “nutrigenetics”, “precision nutrition”, “personalized nutrition”, “nutraceuticals”, “dietary supplements”, “cellular aging”, “biological aging”, “senescence”, “telomere”, “epigenetic age”, and “inflammaging”. Studies published in English, Spanish, or Portuguese were considered eligible.

### 2.5. Study Selection

All records identified through the database searches were imported into Rayyan, a web-based platform for systematic and scoping review management, and duplicate records were removed before screening. Two reviewers independently screened titles and abstracts according to the predefined eligibility criteria. Full-text articles deemed potentially relevant were subsequently assessed for inclusion. Any disagreements arising during the selection process were resolved through discussion and consensus, with consultation from a third reviewer when necessary.

### 2.6. Data Extraction

Data extraction was performed independently by two reviewers using a standardized data collection form. Information extracted from each study included publication characteristics, country of origin, study design, sample size, participant demographics and clinical characteristics, genetic markers evaluated, nutrigenomic strategy employed, type and duration of nutraceutical intervention, comparator group, outcomes assessed, and principal findings related to biomarkers of cellular aging, inflammation, oxidative stress, mitochondrial function, and immunosenescence. No formal risk-of-bias assessment was performed because the purpose of this scoping review was to map the available evidence rather than evaluate intervention efficacy.

### 2.7. Data Synthesis

The extracted data were synthesized descriptively through tabular and graphical presentation, graphs and narrative summary. Studies were categorized according to participant characteristics, nutrigenomic approach, type of nutraceutical intervention, and aging-related outcomes assessed. Given the anticipated heterogeneity in study designs, populations, interventions, and outcome measures, no quantitative meta-analysis was planned. Instead, the review aimed to map the current landscape of evidence, identify methodological trends, and highlight knowledge gaps that may guide future research on precision nutraceutical interventions for healthy aging and longevity.

To characterize the degree of precision-nutrition implementation, studies were further classified using a review-specific translational framework developed for the purposes of evidence synthesis. Tier A comprised interventions in which genetic, omic, metabolic, inflammatory, or deficiency-related information was used prospectively to personalize or stratify the intervention. Tier B comprised standardized interventions in which nutrigenomic, transcriptomic, epigenetic, metabolomic, or biomarker analyses were used to characterize differential responses or identify potential responder profiles, without prospectively determining individualized intervention allocation. Tier C comprised exploratory molecular-response studies in which the intervention was not individualized, but the study provided mechanistic evidence potentially relevant to biomarker discovery or future stratification. Accordingly, Tier A was considered the most direct representation of precision nutraceutical implementation, whereas Tier B and Tier C were retained because they provide translational and mechanistic evidence relevant to the identification of responder profiles, biomarkers, and mechanisms that may support future precision nutraceutical strategies. Tier B and Tier C studies were therefore not interpreted as evidence of clinical efficacy of individualized nutraceutical allocation.

These tiers were intended to describe the degree of personalization and translational maturity of the evidence and should not be interpreted as a hierarchy of methodological quality, risk of bias, or certainty of evidence.

## 3. Results

### 3.1. Selected Studies

**Figure 1:**
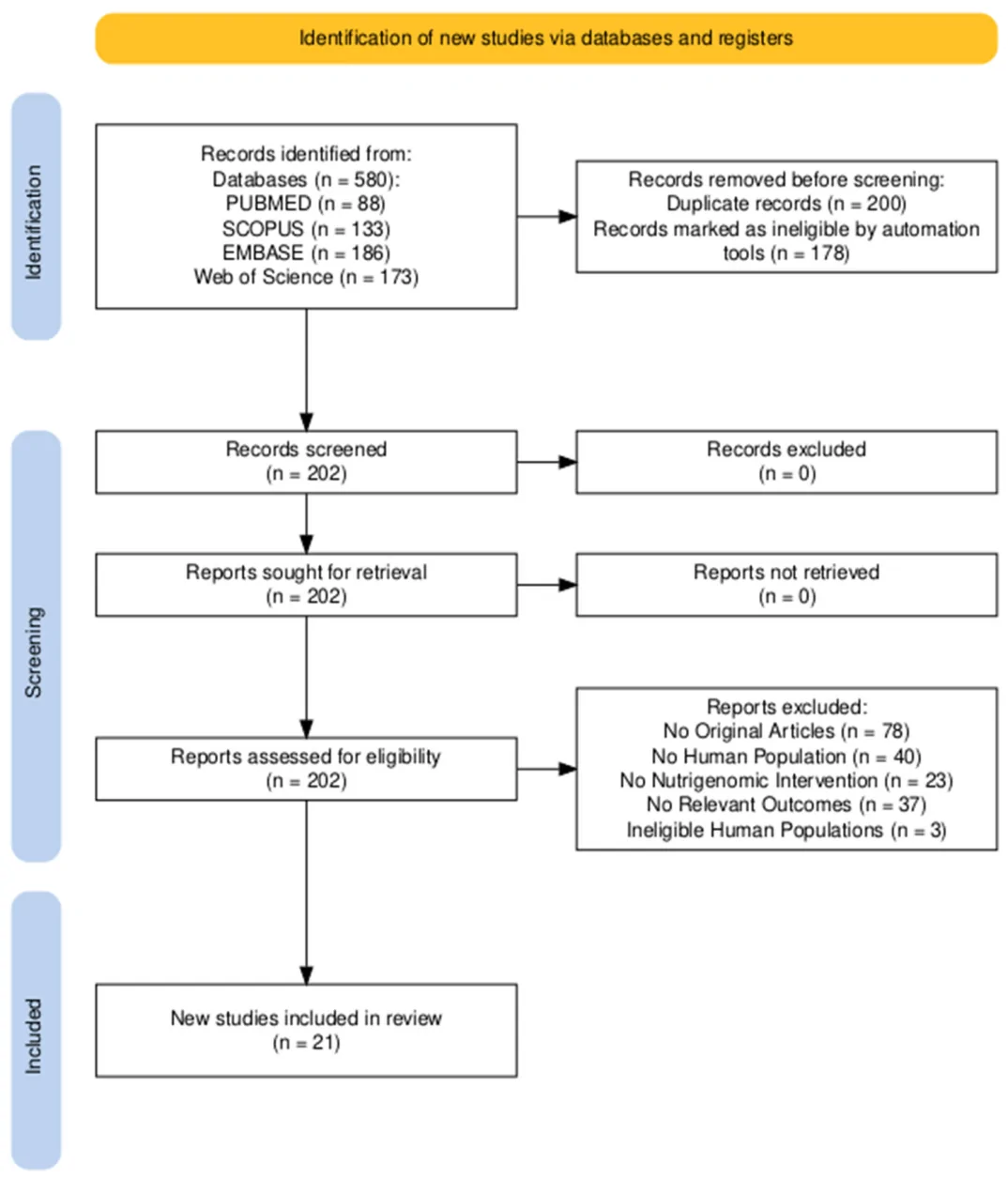
PRISMA 2020 flow diagram of study identification, screening, and selection for inclusion in the scoping review. The diagram summarizes the search results, screening process, reasons for exclusion at the full-text stage, and the final inclusion of 21 studies evaluating nutrigenomic-based personalized nutraceutical interventions and aging-related outcomes. Figure elaborated using the PRISMA Flow Diagram Shiny app v1.0 [19].

A total of 580 records were identified across all information sources (Figure 1). After removing 200 duplicate records, 380 studies remained for title and abstract screening. During this initial screening phase, 178 articles were excluded because they did not meet the predefined eligibility criteria.

The remaining 202 full-text articles were assessed for eligibility through manual review. Of these, 78 studies were excluded because they were not original research articles, 40 were excluded because they were not conducted in human populations, 23 did not include a nutrigenomic-based intervention, 37 did not report outcomes relevant to the review objectives, and 3 involved ineligible human populations. Following the selection process, the remaining studies were included in the final qualitative synthesis. The included studies represented heterogeneous clinical populations, ranging from generally healthy adults to participants with obesity, metabolic disorders, metabolic liver disease, inflammatory conditions, cancer, or other chronic health conditions. Clinical and metabolic characteristics were considered when interpreting intervention responses and are detailed in Supplementary Table S1.

### 3.2. Precision Nutrition and Multi-Omic Stratification Approaches

Precision nutrition applied to nutraceuticals recognizes that the response to an intervention is determined not only by the administered compound but also by the individual’s genetic, metabolic, epigenetic, and phenotypic profile. Studies within this field demonstrate a transition from approaches focused on specific polymorphisms toward multi-omic models integrating metabolomics, proteomics, inflammatory biomarkers, body composition, and digital platforms. Such stratification is particularly relevant to healthy aging, as chronic inflammation, oxidative stress, and metabolic dysfunction exhibit substantial interindividual variability and may strongly influence the efficacy of personalized interventions.

The PREVENTOMICS e-commerce study provides a notable example of multi-omic stratification in personalized nutrition. Calderón-Pérez et al. (2024), in a randomized 21-week follow-up involving 193 adults, found that adherence to the Mediterranean diet improved similarly with personalized and general recommendations, with no significant between-group differences in dietary behavior or general health parameters after adjustment for multiple comparisons. Nevertheless, metabotype-directed recommendations selectively improved composite biomarker scores related to carbohydrate metabolism, oxidative stress, microbiota-derived metabolism, and inflammation, suggesting mechanistic benefits that did not translate into demonstrated clinical superiority [20].

Studies examining functional genetic variants provide an additional level of stratification in precision nutrition. In a 1.5-year randomized intervention involving 188 healthy Finnish adults, Leskinen et al. (2021) evaluated whether disclosure of APOE ε4 carrier status enhanced the response to standardized dietary and lifestyle counseling. Across the overall study population, counseling improved dietary fat quality by 5.3%, increased plasma omega-3 fatty acids by 7.3%, reduced the consumption of high-fat and high-sugar foods from 7.3 to 6.5 portions per week, and decreased total and LDL cholesterol by 4.3% and 6.1%, respectively. However, participants who carried APOE ε4 were not markedly more responsive than the other groups, indicating that genotype disclosure alone provided limited additional benefit beyond general lifestyle guidance [21].

Similarly, Pokushalov et al. (2024) conducted a 180-day randomized, double-blind, placebo-controlled trial in 112 adults with a BMI ≥ 25 kg/m^2^ carrying at least one minor allele in FTO, LEP, LEPR, or MC4R. Compared with placebo, supplementation with glucomannan, inulin, and psyllium, combined with lifestyle counseling, produced greater reductions in body weight (−7.3% vs. −2.4%; between-group difference, −4.9%), BMI, fat mass, and visceral fat rating. Among participants receiving the supplement, homozygous minor-allele carriers experienced greater weight loss than those with mixed allele profiles (−9.4% vs. −5.6%). However, gastrointestinal adverse events were common in the intervention group (74.6%), although their frequency did not differ significantly between genetic subgroups. These findings suggest a potential genotype-related difference in response, but the absence of a genetically unselected comparison group limits conclusions regarding the predictive utility of these variants [22].

Kanoni et al. (2021) extended nutrigenetic stratification to a genome-wide analysis of treatment response in patients with non-alcoholic fatty liver disease (NAFLD) [23]. In a secondary analysis of the MAST4HEALTH randomized, double-blind, placebo-controlled trial, 98 adults with obesity and NAFLD received mastiha (2.1 g/day), a natural resin derived from Pistacia lentiscus, or placebo for six months. Mastiha did not significantly modify most inflammatory or antioxidant biomarkers in the overall population. However, among participants with severe obesity (BMI > 35 kg/m^2^), total antioxidant status was significantly higher with mastiha than with placebo (2.011 vs. 1.824 mmol/L; β = 0.626, *p* = 0.008). Genome-wide analyses also identified several significant gene-by-mastiha interactions involving glutathione peroxidase activity, total antioxidant status, IL-6, IL-10, and TNF-α gene expression. These findings suggest that genetic background may influence the redox-inflammatory response to mastiha; however, the small sample size and lack of independent validation warrant cautious interpretation [23].

Beyond genetic and epigenetic approaches, inflammatory biomarkers have been investigated as accessible tools for disease stratification and precision nutrition. Using data from the two-year Fatty Liver in Obesity (FLiO) randomized trial, Mogna-Pelaez et al. (2024) evaluated 98 adults with metabolic dysfunction-associated steatotic liver disease (MASLD) assigned to either an American Heart Association-based diet or a Mediterranean-style FLiO diet, together with 45 controls [24]. A multivariable model combining leptin, adiponectin, M30, and LECT2 showed high discriminatory performance for MASLD across all assessment points, with AUROC values ranging from 0.952 to 0.995. Moreover, a baseline inflammatory score derived from chemerin, leptin, LECT2, and M65 identified a preferred dietary strategy for achieving greater weight loss in 55% of participants. Although these findings support the potential use of inflammatory biomarkers for dietary stratification, the internally derived model requires external validation before clinical implementation [24].

In a complementary mechanistic approach, Marquez-Paradas et al. (2026) conducted a randomized crossover pilot study in 10 young healthy adults who completed four interventions involving isoenergetic emulsions enriched in saturated fatty acids, monounsaturated fatty acids, omega-3 long-chain polyunsaturated fatty acids, or a fat-free control [25]. Extracellular vesicle concentration and mean diameter remained unchanged across meals and postprandial time points. However, dietary fat composition modified their surface-marker profile and inferred cellular origin: MUFA intake increased CD14^+^ monocyte-associated vesicles, omega-3 fatty acids enhanced vesicles bearing endothelial- and T-cell-related markers during the late postprandial phase, whereas saturated fat reduced several lineage-specific markers. These findings suggest that extracellular vesicle phenotyping may capture acute immunometabolic responses to dietary fat; however, the small sample size, young study population, and exploratory significance threshold limit their relevance to precision nutrition and healthy aging [25].

The included evidence spanned different degrees of precision-nutrition implementation (Supplementary Table S1). Tier A reports prospectively incorporated individual genetic, multi-omic, metabolic, inflammatory, or deficiency-related information into the intervention strategy, whereas Tier B and Tier C reports predominantly used molecular or phenotypic information to characterize response, identify potential responder subgroups, or explore mechanisms without prospectively assigning individualized nutraceutical interventions. Therefore, the latter tiers were interpreted as translational or hypothesis-generating evidence rather than as direct evidence of clinical effectiveness of personalized nutraceutical prescription.

Collectively, these studies suggest that responses to precision nutrition interventions are shaped not only by the intervention itself but also by interindividual variation in genetic background, metabotype, body composition, baseline inflammatory status, and other molecular and phenotypic characteristics. However, the evidence remains heterogeneous and largely exploratory, and few stratification models have undergone external validation. Although subgroup-specific responses have been reported, the clinical utility of multi-omic and biomarker-based approaches for guiding personalized nutraceutical interventions remains uncertain.

### 3.3. Biological Aging Biomarkers and Epigenetic Responses

Human intervention studies have begun to examine whether nutraceuticals and functional foods can modify molecular and cellular processes associated with aging. The studies grouped under this theme extended beyond conventional clinical outcomes to assess DNA methylation, telomere-related measures, oxidative DNA damage, inflammatory signaling, endothelial dysfunction, apoptosis, immunosenescence, and gene-expression profiles. However, most of these outcomes are mechanistically related to aging rather than validated measures of biological age. Evidence based on epigenetic clocks, longitudinal telomere dynamics, or composite biological-age algorithms remains scarce. Accordingly, this literature should primarily be interpreted as evidence of modulation of aging-related pathways rather than as proof that nutraceutical interventions slow biological aging or extend longevity.

Notably, none of the 21 included reports measured biological age using a validated aging clock or composite biological-age algorithm. One study (1/21; 4.8%) assessed telomere-related biomarkers, including telomere length and the proportion of short telomeres, together with a CD8^+^/CD28^−^ T-cell immunosenescence phenotype [26]; however, these measures were not used to derive a validated estimate of biological age. The remaining 20 reports relied predominantly on indirect molecular, metabolic, inflammatory, or physiological biomarkers associated with aging. No study applied a validated DNA methylation-based epigenetic clock, a composite biological-age algorithm, or canonical cellular senescence markers such as p16^INK4A or p21. One additional study assessed intervention-related changes in DNA methylation at individual CpG sites but did not estimate epigenetic age [27]. Thus, although several studies evaluated molecular processes associated with aging, none provided a validated multidimensional estimate of biological age or demonstrated biological-age deceleration.

Da Mota et al. (2026) examined whether methyl-donor supplementation altered adipose-tissue DNA methylation in premenopausal women with inactive systemic lupus erythematosus. Participants received folic acid (400 μg/day) and vitamin B12 (2000 μg/day), or placebo, for 12 weeks and were stratified according to nutritional status. In normal-weight participants, supplementation was associated with 120 differentially methylated CpG sites across 53 genes, including 74 hypermethylated and 46 hypomethylated sites, whereas no significant methylation changes were detected in participants with excess body weight [27]. The affected genes were related to autoimmunity, inflammatory metabolism, obesity, and metabolic regulation. These results suggest that nutritional status may influence epigenetic responsiveness to methyl-donor micronutrients. Nevertheless, the absence of changes in participants with excess body weight does not establish that adiposity or inflammation caused epigenetic resistance. Moreover, the direction, functional consequences, and clinical relevance of the methylation changes remain uncertain, and the study did not assess epigenetic age or determine whether the observed modifications were beneficial [27].

Roitman et al. (2021) reported changes in telomere-related and immunosenescence markers following three and six months of supplementation with OYOX, a commercial product described by the authors as an activator of SIRT1, SIRT3, and SIRT6 [28]. The study reported a reduction in the proportion of short telomeres and senescent CD8^+^/CD28^−^ cytotoxic T cells, together with improvements in glycated hemoglobin, total cholesterol, LDL cholesterol, and homocysteine. These outcomes are potentially relevant because they encompass telomere biology, immunosenescence, and cardiometabolic regulation. However, sirtuin abundance or activity was not directly measured, and the study provided insufficient information regarding participant allocation, group-specific sample sizes, individual-level results, variability estimates, and statistical procedures. The magnitude of the reported telomere changes over a relatively short period is also biologically unexpected. Consequently, these findings should be regarded as highly preliminary and should not be interpreted as evidence of biological-age reversal [26].

Lorenzetti et al. (2023) evaluated fermented papaya preparation (FPP^®^) in an interim analysis of an ongoing approximately 20-month double-blind trial involving 78 clinically stable, community-dwelling adults aged 60–75 years [29]. Participants received either FPP^®^ or an antioxidant mixture, and results from up to 11 months of follow-up were reported. Compared with the antioxidant mixture, FPP^®^ transiently increased plasma inducible nitric oxide synthase at two and six months, reduced asymmetric dimethylarginine at six and eleven months, and maintained lower proportions of apoptotic peripheral blood mononuclear cells during the later assessments. Improvements were also reported in SF-36 domains, particularly physical functioning, general health, and mental health. By contrast, urinary 8-OHdG did not change significantly in the overall population, although exploratory improvements were observed in participants older than 72 years or with a BMI above 27 kg/m^2^ in both intervention groups. These findings suggest potential effects on nitric-oxide regulation, endothelial dysfunction, cellular apoptosis, and functional health, but they do not constitute evidence of epigenetic rejuvenation or deceleration of biological age [29].

Gene-expression studies provide complementary evidence that dietary bioactive compounds can modify molecular pathways associated with oxidative defense, metabolism, and cellular homeostasis. Gualtieri et al. (2023) conducted a randomized crossover trial in 24 healthy adults comparing a Mediterranean diet alone with the same diet supplemented for two weeks with 250 mL/day of mixed apple–bergamot juice [30]. Supplementation increased blood expression of Peroxisome Proliferator-Activated Receptor γ (PPARγ), Superoxide dismutase (SOD1), VDR, and MIF, whereas catalase (CAT), chemokine C-C motif ligand 5 (CCL5), and Nuclear Factor Kappa B Subunit 1 (NFKB1) remained unchanged. Increased PPARγ, SOD1, and VDR expression is consistent with modulation of metabolic regulation, antioxidant defense, and vitamin D signaling. However, the interpretation of the increase in Macrophage Migration Inhibitory Factor (MIF) is less straightforward because this cytokine has pleiotropic and potentially pro-inflammatory functions. Therefore, the small sample and brief intervention support a mechanistic interpretation but do not establish sustained functional or anti-aging benefits [30].

In the randomized crossover MiBLEND study, DeBenedictis et al. (2024) increased fruit and vegetable intake from 50 to 450 g/day using blends enriched in flavonoids, anthocyanins, carotenoids, glucosinolates, or combinations of these phytochemical classes [28]. Across the interventions, increased fruit and vegetable consumption improved plasma antioxidant capacity, resistance to experimentally induced DNA damage, and retinal arteriolar dilation, although the magnitude and pattern of response varied according to the phytochemical composition of the blends. Transcriptomic analyses identified changes in pathways related to DNA repair, cell-cycle regulation, transcription, replication, and signal transduction. These findings support the capacity of phytochemical-rich foods to induce molecular and phenotypic plasticity. Nevertheless, the outcomes represent intermediate indicators of genomic protection and vascular function rather than validated biomarkers of biological age [28].

Teixeira et al. (2023) conducted a 12-week randomized, double-blind, placebo-controlled pilot trial in 28 patients with relapsing–remitting multiple sclerosis [31]. A low-dose formulation containing guaraná, selenium, and L-carnitine reduced plasma oxidized DNA, measured using 8-hydroxy-2′-deoxyguanosine, and shifted circulating cytokine profiles toward a less inflammatory profile, with reductions in IL-1β, IL-6, TNF-α, and IFN-γ and an increase in IL-10. In contrast, the intervention did not produce consistent modulation of NLRP3, CASP-1, or the cytokine-related genes examined. These findings indicate that changes in circulating redox-inflammatory markers can occur without a coordinated transcriptomic response. Given the small sample, the exploratory nature of the study, and the absence of direct biological-age measurements, the results should be interpreted as preliminary evidence of molecular protection rather than evidence of biological-aging deceleration [31].

Collectively, these studies indicate that nutraceuticals and phytochemical-rich foods can modify molecular processes relevant to aging, including DNA methylation, oxidative DNA damage, inflammatory signaling, endothelial dysfunction, apoptosis, immunosenescence, and gene expression. However, the evidence is heterogeneous and dominated by small or short-term studies that rely predominantly on surrogate endpoints. Only isolated studies assessed telomere-related or epigenetic outcomes, and none demonstrated a validated reduction in biological age. The current literature therefore supports the modulation of aging-related biological pathways but does not establish that nutraceutical interventions decelerate biological aging. Future trials should recruit clearly age-defined populations, prespecify validated biological-age measures, incorporate epigenetic clocks and robust cellular-senescence markers, and determine whether molecular changes are accompanied by sustained improvements in physical function, morbidity, and other clinically meaningful outcomes.

### 3.4. Redox-Inflammatory and Immunometabolic Regulation

Redox-inflammatory and immunometabolic regulation represents a central axis for understanding the potential of nutraceuticals beyond their classical antioxidant effects. The evidence reviewed in this topic indicates that bioactive compounds, including polyphenols, omega-3 fatty acids, botanical extracts, micronutrients, and functional foods, can modulate interconnected biological networks involving oxidative stress, chronic low-grade inflammation, lipid metabolism, immune function, DNA damage, inflammatory cell death, vascular signaling, and intercellular communication. This perspective is particularly relevant in conditions such as obesity, metabolic liver disease, autoimmunity, vascular aging, and cardiometabolic risk, in which persistent inflammation and redox imbalance serve as common drivers of disease progression. Consequently, the value of these studies lies not merely in demonstrating changes in isolated biomarkers but in identifying functional response patterns that help explain how nutraceutical interventions may restore immunometabolic homeostasis.

In obesity, studies investigating Nigella sativa (black seed or black cumin), a medicinal plant containing bioactive compounds such as thymoquinone, provide complementary evidence on the interface between inflammation, adipogenesis, and adipose metabolism. In a randomized, double-blind, placebo-controlled crossover trial in women with overweight or obesity, Razmpoosh et al. (2023) showed that Nigella sativa oil supplementation reduced tumor necrosis factor alpha (TNF-α) at both the transcriptional and serum levels and increased peroxisome proliferator-activated receptor gamma (PPAR-γ), adiponectin, and adiponectin receptor 1 (AdipoR1), biomarkers related to adipogenesis, metabolic regulation, and inflammatory control [32]. In a complementary analysis, Razmpoosh et al. (2024) reported reductions in both gene expression and serum levels of interleukin-1β (IL-1β), interleukin-6 (IL-6), and leptin, as well as a statistically significant but small reduction in serum insulin [33]. Collectively, these findings suggest that Nigella sativa oil may help shift obesity-related inflammatory signaling toward a more regulated immunometabolic profile. Nevertheless, these results should be interpreted as preliminary mechanistic evidence owing to the small sample size, crossover design, observed carryover effects for some serum outcomes, restriction to women, and predominant use of surrogate molecular and circulating biomarkers [34].

Omega-3 fatty acids constitute an important model of immunometabolic regulation because of their ability to modulate both the active resolution of inflammation and lipid metabolism. Torres-Vanegas et al. (2025) demonstrated that a diet with a controlled omega-6/omega-3 ratio, supplemented with eicosapentaenoic acid (EPA) and docosahexaenoic acid (DHA), increased resolvin D1 and interleukin-10 (IL-10) while reducing interleukin-6 (IL-6) and monocyte chemoattractant protein-1 (MCP-1) in adults with obesity, suggesting a shift toward a more pro-resolving and less inflammatory phenotype [35]. From a precision nutrition perspective, Medoro et al. (2025) showed that the incorporation of EPA and DHA after fish oil supplementation may vary according to genetic background, particularly polymorphisms in the elongation of very long-chain fatty acids protein 2 gene (ELOVL2), with carriers of the minor ELOVL2 allele showing a greater increase in EPA and the Omega-3 Index. In contrast, no relevant differential response was observed for the fatty acid desaturase 1 gene (FADS1) polymorphism evaluated [36]. Complementarily, Michielsen et al. (2022), using plasma metabolomics in adults with overweight or obesity, showed that fish oil modifies the lipidome beyond total triglycerides by increasing unsaturated lipid species and reducing certain saturated triglyceride species. Together, these findings indicate that the immunometabolic effects of omega-3 fatty acids depend not only on the administered dose but also on the individual’s capacity to incorporate EPA and DHA and remodel the lipid profile toward potentially less atherogenic and less inflammatory signatures [33].

In autoimmune disease, Teixeira et al. (2023) provide a complementary example of redox-inflammatory modulation. In patients with relapsing–remitting multiple sclerosis, a guaraná, selenium, and L-carnitine-enriched coffee formulation reduced oxidized DNA and pro-inflammatory cytokines while increasing IL-10. However, the intervention did not produce robust downregulation of inflammatory pathway genes such as NLRP3 and CASP1, suggesting that changes in circulating oxi-inflammatory biomarkers may occur without parallel transcriptomic suppression of inflammatory signaling [31].

Metabolic liver disease provides a clinically relevant example of the interplay between inflammation, adipose dysfunction, and hepatocellular injury. In the FLiO trial, Mogna-Pelaez et al. (2024) showed that inflammatory and adipokine-related biomarkers can reflect MASLD activity and support treatment customization. Rather than relying only on imaging or complex omics, the study suggests that accessible markers linked to adiposity, apoptosis, and hepatic inflammation may help characterize redox-inflammatory heterogeneity and guide dietary strategies in MASLD [24]. In contrast, Hasankhani et al. (2025) evaluated oleoylethanolamide supplementation in adults with obesity and non-alcoholic fatty liver disease receiving a personalized calorie-restricted diet, but found no significant between-group changes in the expression of pyroptosis-related genes, including TLR4, MyD88, TRIF, NLRP3, caspase-1, caspase-8, IL-1β, and IL-18, or in serum lipopolysaccharide-binding protein (LBP) levels [37]. This negative finding is important because it suggests that previously reported metabolic improvements with OEA do not necessarily translate into detectable modulation of specific inflammatory cell-death pathways, underscoring the need to align biomarker selection with the expected mechanism of action [37].

Kanoni et al. (2021) provide a relevant example of redox-inflammatory modulation in metabolic liver disease, showing that mastiha supplementation in patients with NAFLD produced subgroup-specific antioxidant effects and nutrigenetic interactions involving inflammatory and antioxidant biomarkers such as IL-6, IL-10, and glutathione peroxidase. This supports the notion that genetic background may condition the immunometabolic response to nutraceutical interventions [23].

Polyphenol- and phytochemical-rich foods further support the concept that nutraceutical effects extend beyond direct antioxidant activity. As shown by Gualtieri et al. (2023) and DeBenedictis et al. (2024), these interventions may modulate antioxidant defense, metabolic signaling, DNA damage protection, vascular function, and immune-related transcriptional pathways [28,30]. In this context, their relevance lies not in demonstrating validated anti-aging effects, but in showing that dietary bioactives can influence adaptive redox-inflammatory networks and cellular repair responses.

Functional immunological and vascular outcomes further broaden this redox-inflammatory perspective. Lackner et al. (2022) reported tolerability-dependent immunomodulatory effects of aronia juice polyphenols, including changes in regulatory T-cell dynamics and CD4^+^ T-cell activation [38]. Marquez-Paradas et al. (2026) showed that dietary fat quality acutely modifies the phenotype and cellular origin of circulating extracellular vesicles during the postprandial period [25]. Similarly, Lorenzetti et al. (2023) found that fermented papaya preparation influenced endothelial nitric-oxide-related markers, PBMC apoptosis, and quality-of-life domains in older adults [29]. These findings suggest that nutraceutical responses may involve coordinated effects on immune-cell regulation, vascular signaling, and intercellular communication.

Finally, Gwenzi et al. (2026) extended this topic toward personalized immunomodulation through correction of a biologically defined micronutrient deficiency. In colorectal cancer patients with low vitamin D status who had undergone surgery within the previous year, personalized vitamin D3 supplementation—consisting of a loading dose tailored to baseline 25(OH)D levels and body mass index, followed by 2000 IU/day for 12 weeks—significantly reduced serum interleukin-6 (IL-6) compared with placebo, whereas changes in interferon gamma (IFN-γ) and matrix metalloproteinase-1 (MMP-1) were not statistically significant [39]. This study is relevant to the redox-inflammatory and immunometabolic framework because it suggests that inflammatory modulation may be enhanced when interventions target an underlying nutritional vulnerability rather than applying uniform supplementation. Its principal contribution is to show that specific micronutrient repletion, when individualized according to baseline biological status, can attenuate systemic inflammatory signaling in a clinically vulnerable population [39].

Collectively, the evidence reviewed in this topic suggests that nutraceuticals and functional foods may modulate key components of the redox-inflammatory and immunometabolic axis, including pro-inflammatory cytokines, pro-resolving mediators, adipokines, antioxidant defense, oxidative DNA damage, lipid metabolism, endothelial function, apoptosis, extracellular vesicle signaling, and biomarkers of hepatic or systemic inflammation. Studies involving Nigella sativa, omega-3 fatty acids, mastiha, polyphenols, phytochemical-rich foods, fermented papaya preparation, and vitamin D3 indicate that nutritional responses should not be interpreted solely as direct antioxidant effects. Rather, they appear to involve coordinated modulation of biological networks linking inflammation, metabolism, immune regulation, cellular communication, and vascular homeostasis.

**Figure 2:**
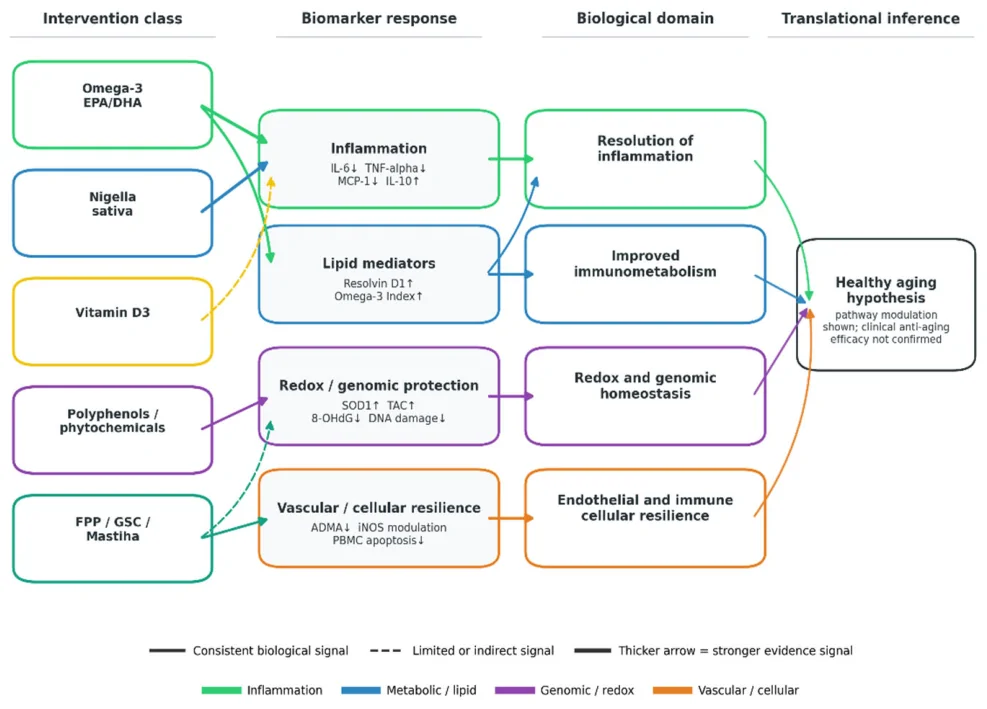
Integrated mechanistic framework of precision nutraceuticals for modulation of aging-related biological pathways. Upward arrows (↑) indicate an increase, activation, or enhancement of the indicated biomarker or biological process, whereas downward arrows (↓) indicate a decrease, inhibition, or attenuation.

Nevertheless, these findings should be interpreted with caution. Most studies were limited by small to moderate sample sizes, relatively short intervention periods, reliance on surrogate biomarkers, and marked heterogeneity in populations, doses, interventions, and outcomes. Moreover, negative or neutral findings, such as the absence of significant changes in pyroptosis-related genes, lipopolysaccharide-binding protein, or selected transcriptomic markers, show that biological plausibility does not always translate into measurable molecular effects. Overall, this evidence reinforces a central conclusion: the efficacy of nutraceutical interventions depends on the baseline biological context, the expected mechanism of action, the suitability of the selected biomarkers, and the identification of responder subgroups. Future studies should integrate redox, inflammatory, metabolomic, transcriptomic, and immunophenotypic biomarkers with clinically meaningful outcomes to determine whether these molecular signals translate into sustained benefits for metabolic, immune, and vascular health. To facilitate the integration of these interconnected mechanisms, Figure 2 summarizes the principal biological pathways modulated by precision nutraceutical interventions and their potential implications for healthy aging.

### 3.5. Clinical Applications in Age-Related Metabolic Disorders

Personalized nutraceutical interventions for age-related metabolic disorders represent an emerging area of clinical research, particularly in conditions characterized by chronic low-grade inflammation, dysfunctional adiposity, insulin resistance, dyslipidemia, and metabolic liver injury. Unlike generalized supplementation approaches, this field examines whether responses to bioactive compounds—such as botanical extracts, omega-3 fatty acids, mastiha, oleoylethanolamide, and liver-support formulations—vary according to the individual’s metabolic, inflammatory, genetic, or biomarker profile. This perspective is especially relevant in obesity, metabolic dysfunction-associated steatotic liver disease (MASLD), and cardiometabolic risk, where biological heterogeneity strongly influences both therapeutic response and the interpretation of intermediate biomarkers. Inflammatory modulation by Nigella sativa is discussed above and is therefore not repeated in detail in this section.

MASLD, formerly encompassed within the spectrum of non-alcoholic fatty liver disease (NAFLD), provides one of the clearest clinical models for personalized nutraceutical and dietary interventions because of its close relationship with obesity, insulin resistance, dyslipidemia, hepatic inflammation, and oxidative stress. Kanoni et al. (2021) showed that six-month mastiha supplementation improved total antioxidant status only in patients with NAFLD and severe obesity, while genome-wide analyses identified gene-by-mastiha interactions involving inflammatory and antioxidant biomarkers, including IL-6, IL-10, glutathione peroxidase, and related metabolic pathways [23]. In the FLiO trial, Mogna-Pelaez et al. (2024) further demonstrated that inflammatory markers can serve not only as non-invasive tools for MASLD screening but also as instruments for treatment customization. Specifically, leptin, adiponectin, M30, and LECT2 showed diagnostic potential across follow-up, while baseline chemerin, leptin, LECT2, and M65 were incorporated into a predictive score to guide dietary strategy selection [24]. Together, these findings suggest that clinical stratification in MASLD may be supported by accessible inflammatory and metabolic biomarkers, in addition to more complex omics approaches.

Evidence from liver-targeted supplementation remains more exploratory. Schauer et al. (2025) evaluated an eight-week multicomponent liver-support regimen containing glutathione, N-acetylcysteine, α-lipoic acid, and other compounds in a small open-label cohort of Austrian adults. The intervention increased urinary D-glucaric acid and mercapturic acids, biomarkers related to glucuronidation and glutathione conjugation pathways, but responses did not differ significantly according to predefined detoxification genotype groups. Therefore, this study supports the feasibility of combining genotyping with urinary biomarkers, but it does not yet establish clinically validated genotype-guided liver supplementation [40].

By contrast, Hasankhani et al. (2025) illustrate the importance of negative mechanistic findings. In adults with obesity and newly diagnosed NAFLD, oleoylethanolamide supplementation combined with a personalized calorie-restricted diet did not significantly modify serum lipopolysaccharide-binding protein or the expression of pyroptosis-related genes in peripheral blood mononuclear cells, including TLR4, MyD88, TRIF, NLRP3, caspase-1, caspase-8, IL-1β, and IL-18 [37]. Although previous analyses from the same research project reported metabolic and hepatic improvements, this specific analysis suggests that clinical or metabolic benefits do not necessarily translate into detectable modulation of selected inflammatory cell-death pathways.

Omega-3 fatty acids are also clinically relevant in age-related cardiometabolic disorders because of their effects on inflammation, lipid metabolism, and cardiovascular risk. Torres-Vanegas et al. (2025) showed that, in adults with obesity, a calorie-restricted diet with an omega-6/omega-3 ratio ≤ 5:1 supplemented with EPA and DHA increased resolvin D1 and IL-10 while reducing IL-6 and MCP-1, suggesting a shift toward a more pro-resolving inflammatory profile [35]. From a nutrigenetic perspective, Medoro et al. (2025) showed that fish oil supplementation increased EPA, DHA, and the Omega-3 Index, with a more favorable response among carriers of the minor ELOVL2 allele, whereas FADS1 showed less consistent differential effects [36]. Complementarily, Michielsen et al. (2022) demonstrated through plasma metabolomics that fish oil modifies the lipidome in adults with overweight or obesity, reducing several saturated triglyceride species and increasing unsaturated lipid species associated with a potentially more favorable cardiometabolic profile [33]. These studies indicate that omega-3 responses are not uniform and may depend on inflammatory status, genetic determinants of fatty acid handling, and baseline lipidomic signatures.

**Figure 3:**
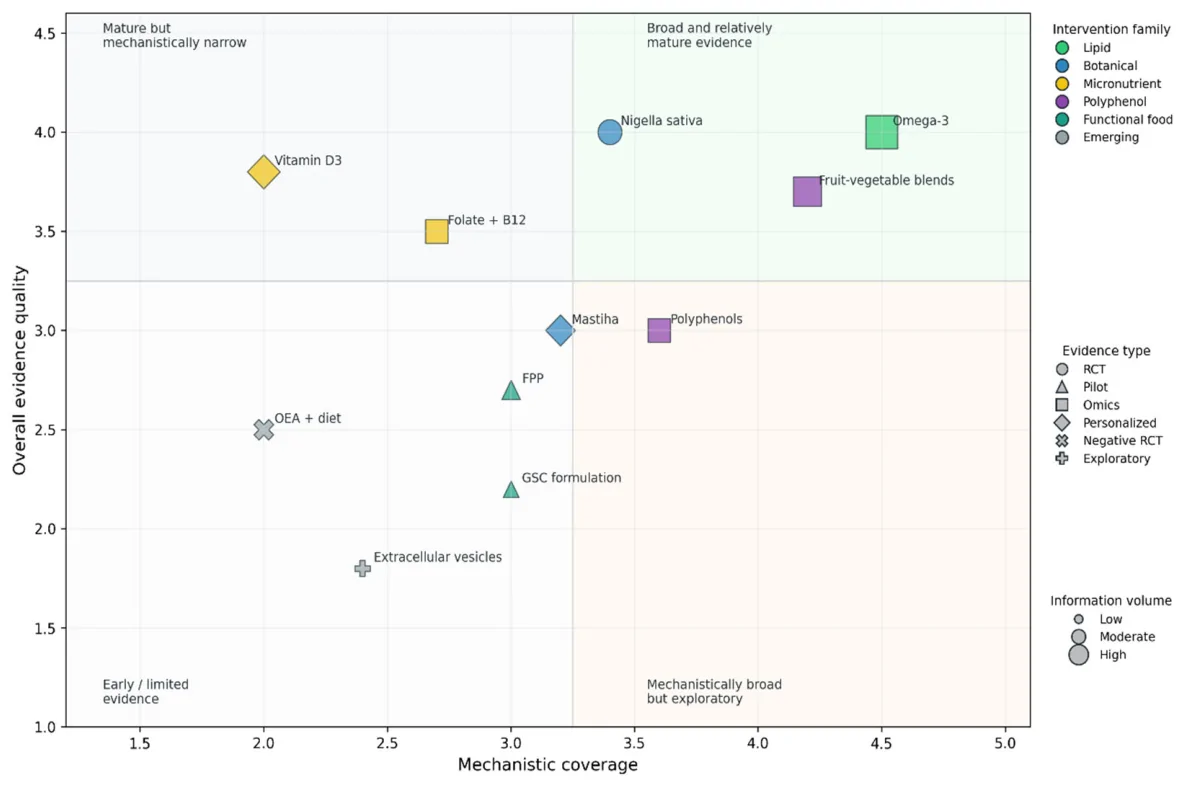
Integrated evidence landscape of precision nutraceutical interventions according to mechanistic coverage and methodological maturity. The *x*-axis represents the relative breadth of biological pathways and molecular biomarkers investigated for each intervention, whereas the y-axis reflects the relative methodological maturity of the available human evidence. Background shading delineates four evidence landscapes: early/limited evidence (lower mechanistic coverage and lower methodological maturity), mechanistically broad but exploratory evidence (higher mechanistic coverage but lower methodological maturity), mature but mechanistically narrow evidence (lower mechanistic coverage but higher methodological maturity), and broad and relatively mature evidence (higher mechanistic coverage and higher methodological maturity). Colors indicate nutraceutical families, marker shapes denote the predominant study design, and symbol size reflects the relative amount of mechanistic information synthesized.

Overall, the evidence reviewed in this section suggests that personalized nutraceutical strategies may have clinical relevance in age-related metabolic disorders by modulating pathways related to inflammation, antioxidant status, lipid metabolism, hepatic injury, inflammatory resolution, and metabolic homeostasis. However, the evidence remains preliminary. Several studies rely on small or moderate sample sizes, short intervention periods, surrogate biomarkers, exploratory omics analyses, or subgroup findings. Moreover, some stratification models did not robustly predict treatment response, and some biologically plausible interventions failed to modify the expected molecular pathways. Therefore, personalized nutraceutical interventions should not be viewed as a universal application of dietary supplements, but as a precision strategy that requires careful identification of responder subgroups, alignment between intervention and mechanism of action, selection of appropriate biomarkers, and validation in larger longitudinal trials with clinically meaningful outcomes. Figure 3 summarizes the relative mechanistic breadth and methodological maturity of the precision nutraceutical interventions identified in this review.

### 3.6. Patient-Relevant Clinical and Functional Outcomes

Patient-relevant clinical and functional outcomes were infrequently assessed across the included evidence. Among the 21 included reports, four (19.0%) reported a patient-relevant efficacy or functional outcome. Calderón-Pérez et al. assessed physical activity using the International Physical Activity Questionnaire (IPAQ), with a decrease observed in the subgroup receiving the inflammation-targeted dietary plan [20]; Leskinen et al. assessed physical activity frequency, without a significant intervention-related improvement [21]; Pokushalov et al. evaluated participant-reported appetite, hunger, fullness, and satiety using visual analogue scales [22]; and Lorenzetti et al. assessed health-related quality of life using the SF-36, including self-reported physical functioning and mental health domains [29]. Several additional reports measured physical activity primarily as a baseline characteristic, covariate, or lifestyle-control variable rather than as an intervention efficacy outcome. Patient-reported gastrointestinal symptoms or tolerability were also reported in some studies as safety outcomes [38]. No included report prospectively assessed objective physical performance, cognitive function, incident age-related disease, hospitalization, mortality, or longitudinal frailty or disability progression. Study-level details, including the distinction between patient-relevant clinical/functional outcomes and intermediate molecular, metabolic, anthropometric, or imaging outcomes, are provided in Supplementary Table S1.

## 4. Discussion

This scoping review mapped the available human evidence on nutrigenomic-based and precision nutraceutical interventions targeting biomarkers related to healthy aging. The main finding is that the field is biologically promising but clinically immature. Across the included studies, nutraceuticals and functional foods were associated with changes in intermediate biomarkers related to inflammation, oxidative stress, lipid metabolism, endothelial function, DNA methylation, immune regulation, apoptosis, extracellular vesicle signaling, and metabolic homeostasis [20,24,25,26,27,28,29,30,31,32,33,34,35,36,37,38,39,40]. However, most studies did not assess validated biomarkers of biological aging, such as epigenetic clocks, longitudinal telomere dynamics, robust cellular senescence markers, or composite biological-age algorithms [6,9]. Therefore, the available evidence should be interpreted as supporting the modulation of aging-related biological pathways rather than demonstrating deceleration of biological aging or rejuvenation.

An important conceptual consideration is the heterogeneity of the term nutraceutical itself. The included literature encompasses concentrated supplements, food-derived preparations, and functional food-based interventions, as well as studies investigating individual bioactive constituents. These categories should not be considered interchangeable. Accordingly, our synthesis distinguishes the intervention or preparation from its constituent bioactive compounds and interprets biological responses in the context of dose, formulation, bioavailability, and individual characteristics.

Regarding our review, a first important observation is the transition from nutrigenetic approaches based on isolated polymorphisms toward broader stratification models integrating genetic, metabolic, inflammatory, and multi-omic information [12,13,15]. The PREVENTOMICS e-commerce study represents one of the most comprehensive examples of this approach, integrating metabolomics, proteomics, genetics, and digital behavioral tools to deliver personalized dietary recommendations [20]. Although personalized recommendations did not show superiority over general Mediterranean diet advice for overall dietary behavior or general health parameters after adjustment for multiple comparisons, metabotype-directed recommendations selectively improved composite scores related to carbohydrate metabolism, oxidative stress, microbiota-derived metabolism, and inflammation [20]. This finding is important because it suggests that the main contribution of multi-omic precision nutrition may not necessarily be immediate clinical superiority, but rather improved biological stratification and identification of mechanistic response patterns. Similarly, the APOE genotype disclosure trial showed that genetic risk information alone produced limited additional benefit beyond standard lifestyle counseling, reinforcing the idea that genetic information must be embedded within sustained behavioral, nutritional, and clinical support to generate meaningful health effects [21].

Omega-3 fatty acids provide a useful model for understanding the clinical relevance of integrating genetics, lipid metabolism, and inflammatory resolution. Fish oil supplementation increased EPA, DHA, and the Omega-3 Index, but the magnitude of incorporation varied according to genetic background, particularly ELOVL2 polymorphisms [36]. Complementary metabolomic evidence showed that fish oil modifies the plasma lipidome beyond conventional triglyceride measures, reducing relatively saturated triglyceride species and increasing several unsaturated lipid species [33]. In adults with obesity, a diet with a controlled omega-6/omega-3 ratio supplemented with EPA and DHA also increased resolvin D1 and IL-10 while reducing IL-6 and MCP-1, indicating a shift toward a more pro-resolving inflammatory profile [35]. Together, these findings suggest that omega-3 responses should not be evaluated only according to the administered dose. Rather, their biological effects appear to depend on the individual capacity to incorporate EPA and DHA, remodel lipid species, and activate specialized pro-resolving pathways [33,34,35].

The evidence also indicates that botanical extracts and phytochemical-rich foods can modulate inflammatory, antioxidant, and metabolic networks, although with varying methodological strength. Trials involving Nigella sativa oil in women with overweight or obesity reported reductions in TNF-α, IL-1β, IL-6, leptin, and insulin, together with increases in PPAR-γ, adiponectin, and AdipoR1 [32,34]. These findings suggest potential modulation of the interface between inflammation, adipogenesis, and metabolic regulation. However, the evidence remains preliminary because these studies were restricted to women, involved modest sample sizes, used surrogate molecular and circulating biomarkers, and reported carryover effects for some outcomes [32,34]. Similarly, mastiha supplementation in patients with NAFLD improved total antioxidant status only in the subgroup with severe obesity and identified several gene-by-mastiha interactions involving inflammatory and antioxidant markers [23]. This finding supports the need to identify responder subgroups, but also illustrates the exploratory nature of current nutrigenetic models.

A central contribution of this review is the distinction between biomarkers associated with aging-related mechanisms and validated measures of biological aging. Several studies assessed DNA methylation, telomere-related markers, oxidative DNA damage, antioxidant gene expression, endothelial function, apoptosis, immunosenescence, and extracellular vesicles [25,26,27,28,29,30,31]. These outcomes are conceptually linked to the hallmarks of aging, including genomic instability, epigenetic alterations, chronic inflammation, altered intercellular communication, mitochondrial dysfunction, and cellular senescence [5,6]. Nevertheless, most of them are not equivalent to biological age. For example, methyl-donor supplementation with folic acid and vitamin B12 modified adipose-tissue DNA methylation in normal-weight women with inactive systemic lupus erythematosus, but not in those with excess body weight [27]. This suggests that nutritional status may influence epigenetic responsiveness, but the study did not assess epigenetic age or determine whether the methylation changes were beneficial [27]. Likewise, fermented papaya preparation influenced nitric-oxide-related endothelial markers, PBMC apoptosis, and quality-of-life domains in older adults, but did not provide evidence of biological-age reversal [29]. Thus, the current evidence supports modulation of aging-related pathways, not confirmed slowing of biological aging.

Epigenomics emerges as a particularly relevant but still underdeveloped domain in precision nutraceutical research. The findings from methyl-donor supplementation in systemic lupus erythematosus suggest that adiposity and baseline inflammatory-metabolic status may condition epigenetic plasticity [27]. This shifts the focus from a simple nutrient-response model toward a more nuanced question: which individuals retain the biological capacity to respond to specific micronutrient interventions? From a precision nutrition perspective, this is highly relevant because the same compound may produce different molecular effects depending on body composition, inflammatory burden, metabolic status, and tissue-specific epigenetic regulation [12,15,27]. However, future studies must go beyond differential methylation sites and incorporate validated epigenetic clocks, gene-expression consequences, functional pathway analyses, and clinical outcomes [6,9].

Redox-inflammatory and immunometabolic regulation was the most consistent biological axis across the included studies. Nutraceuticals and functional foods influenced cytokine signaling, antioxidant defense, pro-resolving mediators, adipokines, endothelial markers, DNA damage, and immune-cell phenotypes [23,24,25,28,29,30,31,32,33,34,35,36,37,38,39]. Teixeira et al. showed that a guaraná, selenium, and L-carnitine-enriched coffee formulation reduced oxidized DNA and pro-inflammatory cytokines in patients with relapsing–remitting multiple sclerosis, although without robust downregulation of inflammatory pathway genes such as NLRP3 or CASP1 [31]. Gualtieri et al. reported increased expression of genes related to antioxidant defense, metabolism, and immune regulation after apple–bergamot juice supplementation [30], while the MiBLEND study showed that phytochemical-rich fruit and vegetable blends improved plasma antioxidant capacity, resistance to DNA damage, retinal microvascular parameters, and transcriptomic pathways related to DNA repair and immune responses [28]. Collectively, these studies suggest that dietary bioactives may act less as isolated antioxidants and more as modulators of adaptive cellular networks [28,30,31].

Studies in metabolic liver disease further highlight the importance of aligning biomarker selection with the expected mechanism of action. In the FLiO trial, inflammatory and adipokine-related markers such as leptin, adiponectin, M30, LECT2, chemerin, and M65 were evaluated as tools for MASLD diagnosis and dietary treatment customization [24]. This supports the clinical utility of accessible biomarkers for stratification, especially in conditions where advanced omics technologies may not be feasible in routine practice. In contrast, oleoylethanolamide supplementation combined with a personalized calorie-restricted diet did not significantly modify serum lipopolysaccharide-binding protein or pyroptosis-related gene expression in adults with obesity and NAFLD [37]. Similarly, a liver-support supplement containing glutathione, N-acetylcysteine, α-lipoic acid, and other compounds increased urinary biomarkers related to glucuronidation and glutathione conjugation, but predefined detoxification-related genetic variants did not robustly predict differential response [40]. These partially positive or negative findings are valuable because they show that biological plausibility does not always translate into measurable modulation of the selected molecular pathway [24,37,40].

Another emerging area is the use of dynamic and functional biomarkers to detect early biological responses to nutritional interventions. Extracellular vesicle phenotyping may capture acute postprandial immunometabolic responses to dietary fat quality, even when vesicle concentration and size remain unchanged [25]. Similarly, aronia juice polyphenols showed tolerability-dependent immunomodulatory effects, including changes in regulatory T-cell dynamics and CD4^+^ T-cell activation [38]. These studies broaden the scope of precision nutraceutical research by suggesting that response may occur through immune-cell behavior, intercellular communication, endothelial signaling, or transient postprandial changes rather than through static circulating biomarkers alone [25,38]. However, these approaches remain exploratory and require validation in larger, older, and metabolically at-risk populations.

Personalized vitamin D3 supplementation in colorectal cancer patients with low vitamin D status provides an important translational lesson: some nutraceutical interventions may be most effective when they correct an underlying biological vulnerability. In this study, vitamin D3 dosing was individualized according to baseline 25(OH)D levels and body mass index, and supplementation significantly reduced IL-6, although IFN-γ and MMP-1 were not significantly modified [39]. This supports the principle that targeted repletion may produce measurable inflammatory benefits in selected populations. However, the cancer-specific context, short duration, and reliance on inflammatory biomarkers limit generalizability to healthy aging populations [39,41].

From a clinical translation perspective, the findings of this review suggest that personalized nutraceutical interventions should evolve toward stepwise models of implementation. First, accessible clinical and biochemical markers, including body composition, lipid profile, glucose metabolism, adipokines, CRP, IL-6, liver markers, vitamin status, and inflammatory phenotype, may help identify individuals most likely to benefit from targeted interventions [24,35,39]. Second, genetic, epigenetic, metabolomic, transcriptomic, or immunophenotypic tools should be applied when there is a clear mechanistic hypothesis and evidence that the selected biomarker predicts or explains treatment response [20,23,25,27,28,36]. This pragmatic approach is important because precision nutrition based exclusively on complex omics technologies may be expensive, difficult to implement, and poorly generalizable in routine clinical practice [12,15,42].

Several limitations of the current evidence must be considered. First, many studies included small samples, pilot designs, short follow-up periods, or surrogate outcomes, limiting causal inference and clinical interpretation. Second, there was substantial heterogeneity in populations, age ranges, health status, interventions, doses, comparators, and biomarkers. Third, some studies were conducted in younger adults or broadly defined adult populations rather than in clearly middle-aged or older adults, reducing their direct applicability to biological aging. Fourth, not all included studies tested truly personalized interventions; some characterized molecular responses to bioactive compounds without genotype-guided or biomarker-guided allocation. Although these studies are relevant to the mapping purpose of a scoping review, they contribute mechanistic rather than definitive personalization evidence. Fifth, most stratification models lacked external validation, limiting their readiness for clinical implementation.

Despite these limitations, this review identifies a clear conceptual direction. Personalized nutraceuticals may have their greatest value when understood as modulators of biological networks rather than as universal supplements with uniform effects. The convergence of findings across inflammation, oxidative stress, lipid metabolism, DNA methylation, endothelial function, apoptosis, genomic protection, extracellular vesicle signaling, and immunometabolism provides substantial biological plausibility [20,21,22,23,24,25,26,27,28,29,30,31,32,33,34,35,36,37,38,39,40,41,42]. However, plausibility is not equivalent to clinical efficacy. Future studies should prioritize adequately powered longitudinal randomized controlled trials with prespecified mechanistic hypotheses, clearly defined responder phenotypes, validated biomarkers of biological aging, standardized biomarker panels, robust adherence assessment, safety monitoring, and clinically meaningful outcomes [6,9,12,15,42].

Future research should also distinguish more clearly among predictive biomarkers, response biomarkers, mechanistic biomarkers, and biomarkers of biological aging. This distinction is essential because the interchangeable use of these terms may lead to overinterpretation. A biomarker that changes after supplementation does not necessarily indicate improved aging biology, and a nutrigenetic association does not necessarily establish clinical utility. To advance the field, studies should integrate molecular biomarkers with functional endpoints such as physical performance, frailty, cardiometabolic events, quality of life, immune resilience, and long-term disease risk [6,9,42]. External validation of stratification models and cost-effectiveness analyses will also be necessary before precision nutraceutical strategies can be incorporated into preventive or clinical practice [12,15,42].

In summary, the evidence reviewed suggests that nutrigenomic-based and precision nutraceutical interventions can modulate biological pathways relevant to healthy aging, especially inflammation, oxidative stress, lipid metabolism, immune regulation, endothelial function, and metabolic homeostasis. However, the field remains in a transitional stage. Current findings support biological plausibility and mechanistic promise, but they do not yet establish robust clinical evidence that personalized nutraceuticals slow biological aging. The next phase of research should move from exploratory biomarker modulation toward validated, clinically meaningful, and implementable precision nutrition strategies.

## 5. Conclusions

Current evidence supports the biological plausibility that personalized nutraceutical interventions can modulate pathways associated with aging, including inflammation, oxidative stress, and immunometabolism. However, robust evidence demonstrating clinically meaningful slowing of biological aging remains lacking. The field is presently in a transitional phase, moving from nutrigenomic promise toward the need for rigorous clinical validation. Future progress will depend on integrating biological precision, clinical feasibility, and methodologically robust evidence to determine which nutraceuticals are effective, in which subgroups, through which mechanisms, and with what tangible impact on healthy aging.

## Data Availability

No new data were created or analyzed in this study. Data sharing is not applicable to this article.

## Supplementary Materials

The following supporting information can be downloaded at: https://www.mdpi.com/article/10.3390/medsci14050508/s1, Table S1: Characteristics of the included studies.
